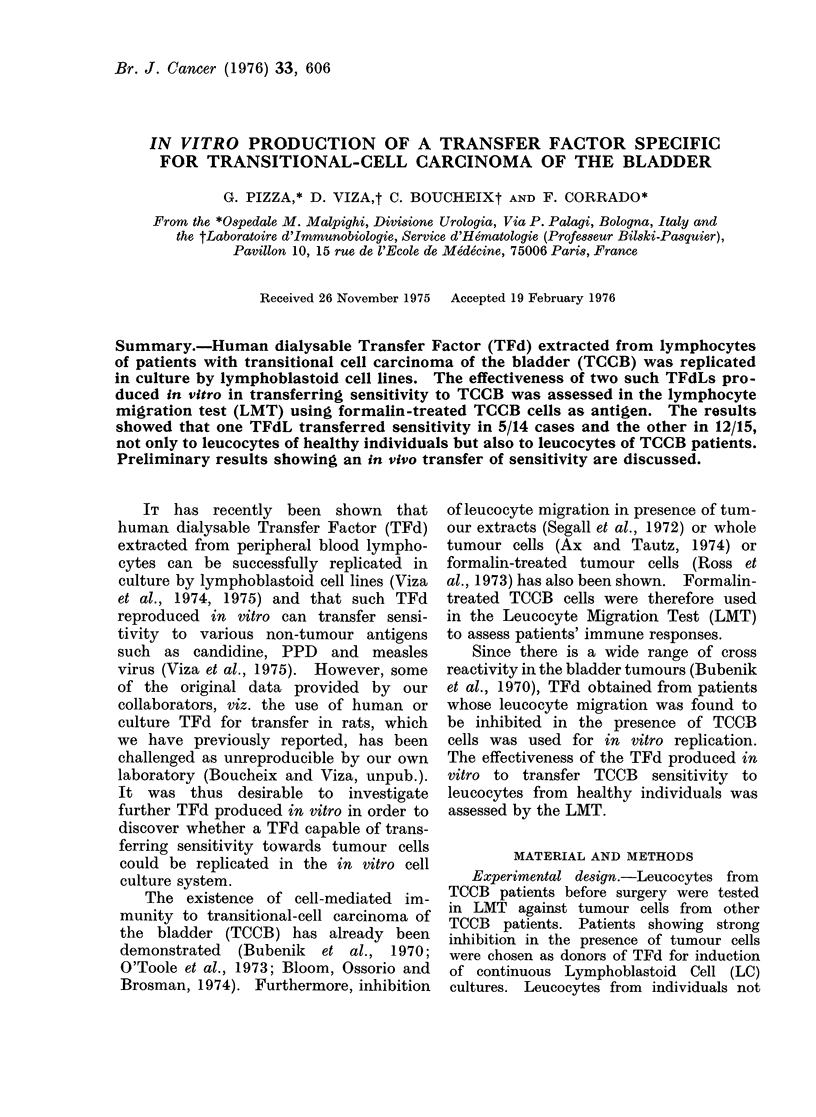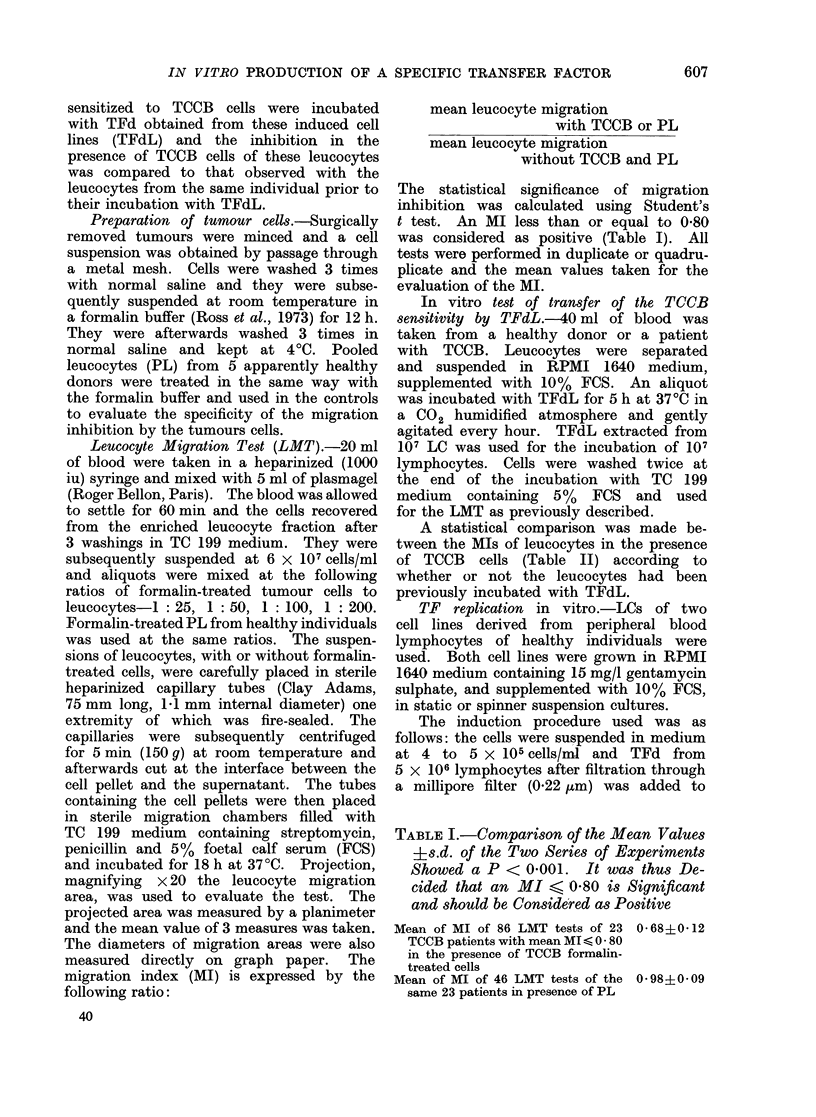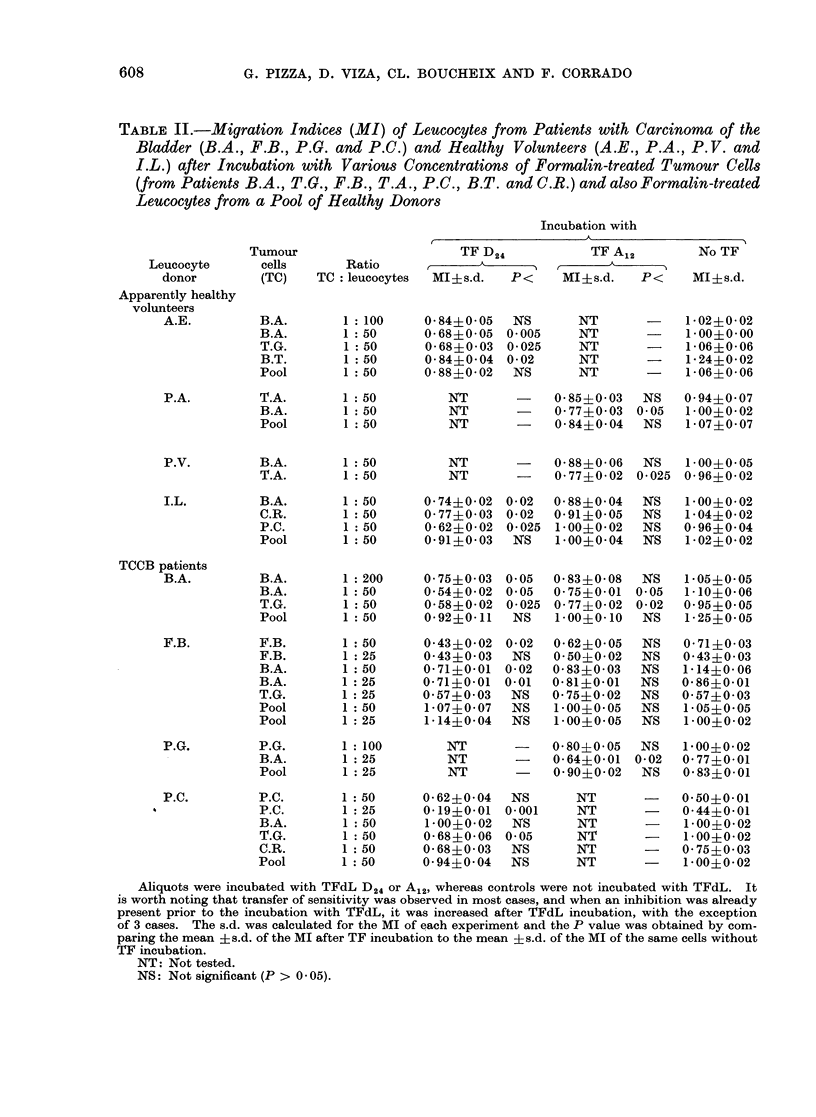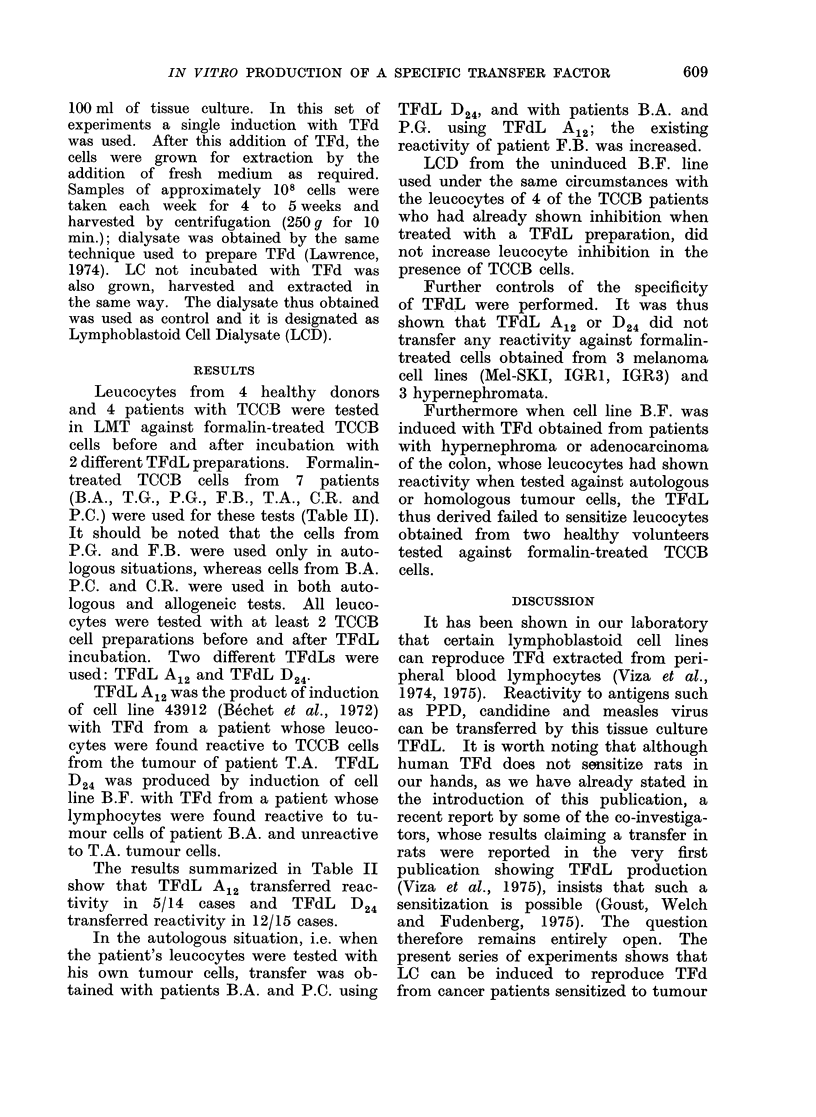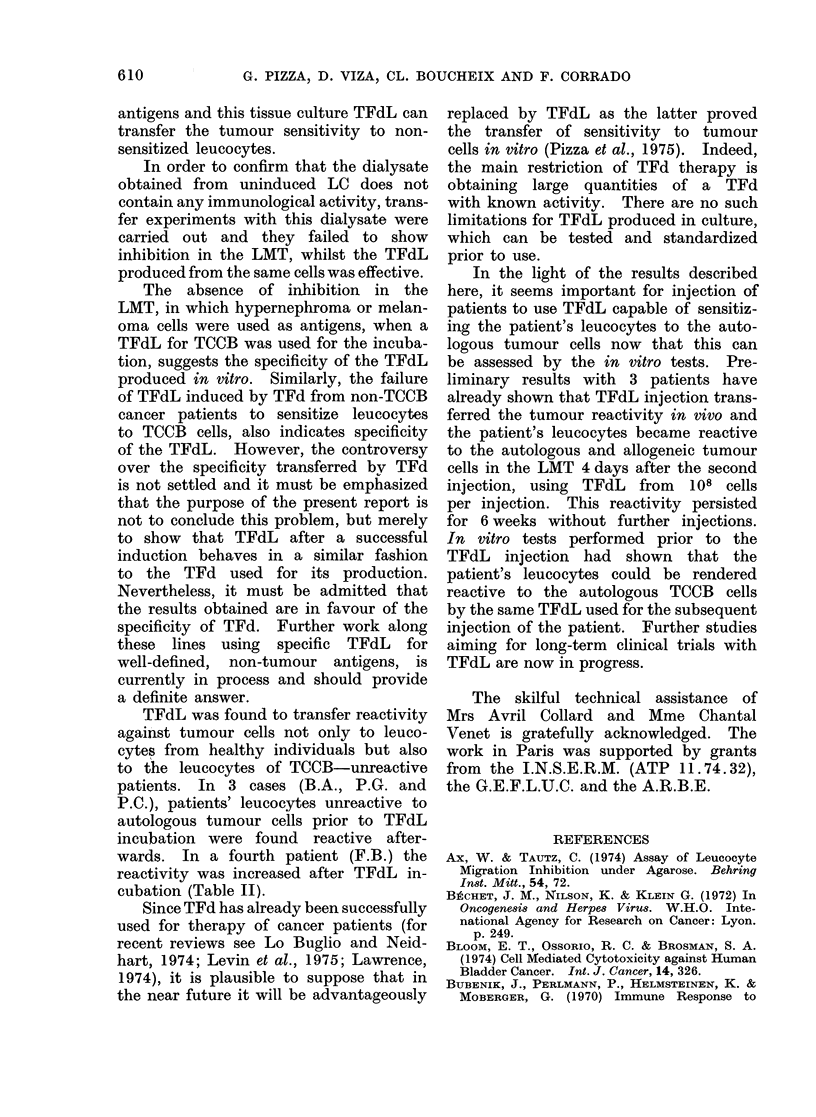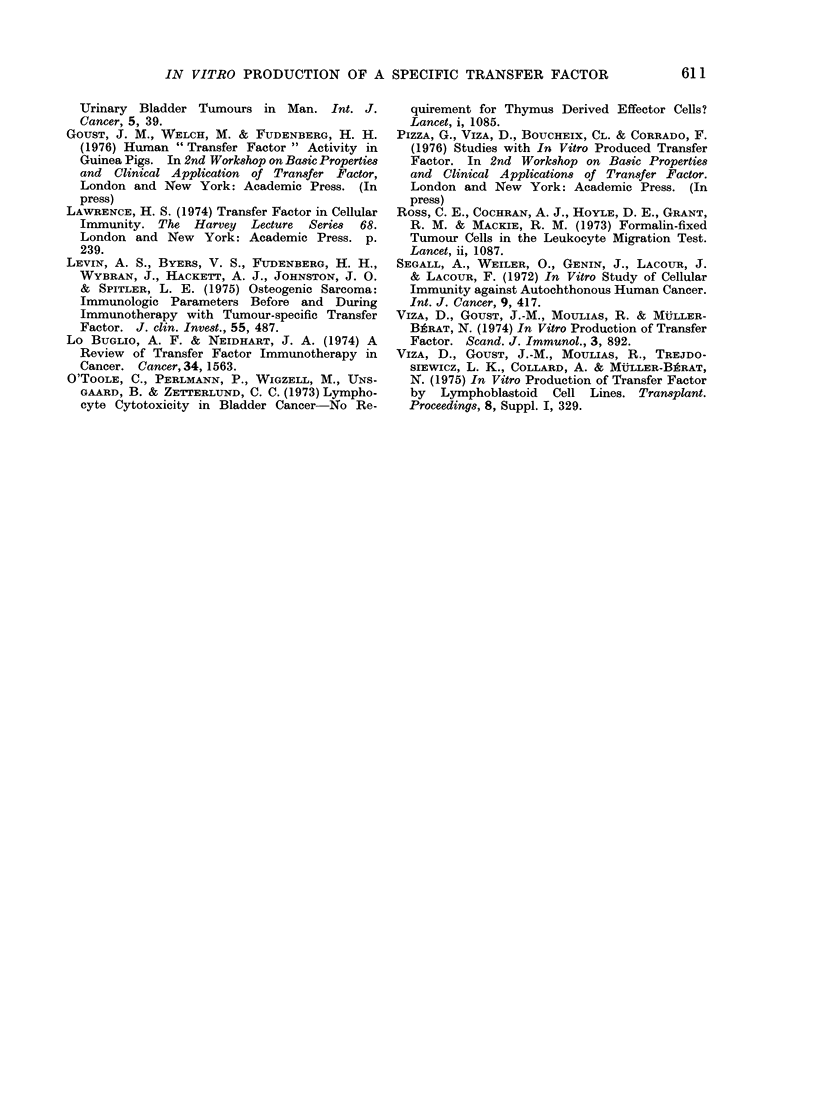# In vitro production of a transfer factor specific for transitional-cell carcinoma of the bladder.

**DOI:** 10.1038/bjc.1976.98

**Published:** 1976-06

**Authors:** G. Pizza, D. Viza, C. Boucheix, F. Corrado

## Abstract

Human dialysable Transfer Factor (TFd) extracted from lymphocytes of patients with transitional cell carcinoma of bladder (TCCB) was replicated in culture by lymphoblastoid cell lines. The effectiveness of two such TFdLs produced in vitro in transferring sensitivity to TCCB was assessed in the lymphocyte migration test (LMT) using formalin-treated TCCB cells as antigen. The results, showed that one TFdL transferred sensitivity in 5/14 cases and the other in 12/15, not only to leucocytes of healthy individuals but also to leucocytes of TCCB patients. Preliminary results showing an in vivo transfer of sensitivity are discussed.


*Br. J. Cancer* (1976) **33**, 606

### *IN VITRO* PRODUCTION OF A TRANSFER FACTOR SPECIFIC FOR TRANSITIONAL-CELL CARCINOMA OF THE BLADDER

G. PIZZA,* D. VIZA,† C. BOUCHEIX† AND F. CORRADO*

*From the *Ospedale M. Malpighi, Divisione Urologia, Via P. Palagi, Bologna, Italy and the †Laboratoire d'Immunobiologie, Service d'Hématologie (Professeur Bilski-Pasquier), Pavillon 10, 15 rue de l'Ecole de Médécine, 75006 Paris, France*

Received 26 November 1975 Accepted 19 February 1976

**Summary.—Human dialysable Transfer Factor (TFd) extracted from lymphocytes of patients with transitional cell carcinoma of the bladder (TCCB) was replicated in culture by lymphoblastoid cell lines. The effectiveness of two such TFdLs produced *in vitro* in transferring sensitivity to TCCB was assessed in the lymphocyte migration test (LMT) using formalin-treated TCCB cells as antigen. The results showed that one TFdL transferred sensitivity in 5/14 cases and the other in 12/15, not only to leucocytes of healthy individuals but also to leucocytes of TCCB patients. Preliminary results showing an *in vivo* transfer of sensitivity are discussed.**

It has recently been shown that human dialysable Transfer Factor (TFd) extracted from peripheral blood lymphocytes can be successfully replicated in culture by lymphoblastoid cell lines (Viza *et al.*, 1974, 1975) and that such TFd reproduced *in vitro* can transfer sensitivity to various non-tumour antigens such as candidine, PPD and measles virus (Viza *et al.*, 1975). However, some of the original data provided by our collaborators, *viz.* the use of human or culture TFd for transfer in rats, which we have previously reported, has been challenged as unreproducible by our own laboratory (Boucheix and Viza, unpub.). It was thus desirable to investigate further TFd produced *in vitro* in order to discover whether a TFd capable of transferring sensitivity towards tumour cells could be replicated in the *in vitro* cell culture system.

The existence of cell-mediated immunity to transitional-cell carcinoma of the bladder (TCCB) has already been demonstrated (Bubenik *et al.*, 1970; O'Toole *et al.*, 1973; Bloom, Ossorio and Brosman, 1974). Furthermore, inhibition of leucocyte migration in presence of tumour extracts (Segall *et al.*, 1972) or whole tumour cells (Ax and Tautz, 1974) or formalin-treated tumour cells (Ross *et al.*, 1973) has also been shown. Formalin-treated TCCB cells were therefore used in the Leucocyte Migration Test (LMT) to assess patients' immune responses.

Since there is a wide range of cross reactivity in the bladder tumours (Bubenik *et al.*, 1970), TFd obtained from patients whose leucocyte migration was found to be inhibited in the presence of TCCB cells was used for *in vitro* replication. The effectiveness of the TFd produced *in vitro* to transfer TCCB sensitivity to leucocytes from healthy individuals was assessed by the LMT.

### MATERIAL AND METHODS

*Experimental design.*—Leucocytes from TCCB patients before surgery were tested in LMT against tumour cells from other TCCB patients. Patients showing strong inhibition in the presence of tumour cells were chosen as donors of TFd for induction of continuous Lymphoblastoid Cell (LC) cultures. Leucocytes from individuals not sensitized to TCCB cells were incubated with TFd obtained from these induced cell lines (TFdL) and the inhibition in the presence of TCCB cells of these leucocytes was compared to that observed with the leucocytes from the same individual prior to their incubation with TFdL.

*Preparation of tumour cells.*—Surgically removed tumours were minced and a cell suspension was obtained by passage through a metal mesh. Cells were washed 3 times with normal saline and they were subsequently suspended at room temperature in a formalin buffer (Ross *et al.*, 1973) for 12 h. They were afterwards washed 3 times in normal saline and kept at 4°C. Pooled leucocytes (PL) from 5 apparently healthy donors were treated in the same way with the formalin buffer and used in the controls to evaluate the specificity of the migration inhibition by the tumours cells.

*Leucocyte Migration Test* (*LMT*).—20 ml of blood were taken in a heparinized (1000 iu) syringe and mixed with 5 ml of plasmagel (Roger Bellon, Paris). The blood was allowed to settle for 60 min and the cells recovered from the enriched leucocyte fraction after 3 washings in TC 199 medium. They were subsequently suspended at $6 \times 10^7$ cells/ml and aliquots were mixed at the following ratios of formalin-treated tumour cells to leucocytes—1 : 25, 1 : 50, 1 : 100, 1 : 200. Formalin-treated PL from healthy individuals was used at the same ratios. The suspensions of leucocytes, with or without formalin-treated cells, were carefully placed in sterile heparinized capillary tubes (Clay Adams, 75 mm long, 1·1 mm internal diameter) one extremity of which was fire-sealed. The capillaries were subsequently centrifuged for 5 min (150 *g*) at room temperature and afterwards cut at the interface between the cell pellet and the supernatant. The tubes containing the cell pellets were then placed in sterile migration chambers filled with TC 199 medium containing streptomycin, penicillin and 5% foetal calf serum (FCS) and incubated for 18 h at 37°C. Projection, magnifying ×20 the leucocyte migration area, was used to evaluate the test. The projected area was measured by a planimeter and the mean value of 3 measures was taken. The diameters of migration areas were also measured directly on graph paper. The migration index (MI) is expressed by the following ratio:

$$\frac{\text{mean leucocyte migration with TCCB or PL}}{\text{mean leucocyte migration without TCCB and PL}}$$

The statistical significance of migration inhibition was calculated using Student's *t* test. An MI less than or equal to 0·80 was considered as positive (Table I). All tests were performed in duplicate or quadruplicate and the mean values taken for the evaluation of the MI.

In vitro *test of transfer of the TCCB sensitivity by TFdL.*—40 ml of blood was taken from a healthy donor or a patient with TCCB. Leucocytes were separated and suspended in RPMI 1640 medium, supplemented with 10% FCS. An aliquot was incubated with TFdL for 5 h at 37°C in a $CO_2$ humidified atmosphere and gently agitated every hour. TFdL extracted from $10^7$ LC was used for the incubation of $10^7$ lymphocytes. Cells were washed twice at the end of the incubation with TC 199 medium containing 5% FCS and used for the LMT as previously described.

A statistical comparison was made between the MIs of leucocytes in the presence of TCCB cells (Table II) according to whether or not the leucocytes had been previously incubated with TFdL.

*TF replication* in vitro.—LCs of two cell lines derived from peripheral blood lymphocytes of healthy individuals were used. Both cell lines were grown in RPMI 1640 medium containing 15 mg/l gentamycin sulphate, and supplemented with 10% FCS, in static or spinner suspension cultures.

The induction procedure used was as follows: the cells were suspended in medium at 4 to $5 \times 10^5$ cells/ml and TFd from $5 \times 10^6$ lymphocytes after filtration through a millipore filter (0·22 $\mu$m) was added to

TABLE I.—*Comparison of the Mean Values ±s.d. of the Two Series of Experiments Showed a $P < 0{\cdot}001$. It was thus Decided that an MI $\leqslant$ 0·80 is Significant and should be Considered as Positive*

| | |
|---|---|
| Mean of MI of 86 LMT tests of 23 TCCB patients with mean MI ≤ 0·80 in the presence of TCCB formalin-treated cells | 0·68±0·12 |
| Mean of MI of 46 LMT tests of the same 23 patients in presence of PL | 0·98±0·09 |

TABLE II.—*Migration Indices (MI) of Leucocytes from Patients with Carcinoma of the Bladder (B.A., F.B., P.G. and P.C.) and Healthy Volunteers (A.E., P.A., P.V. and I.L.) after Incubation with Various Concentrations of Formalin-treated Tumour Cells (from Patients B.A., T.G., F.B., T.A., P.C., B.T. and C.R.) and also Formalin-treated Leucocytes from a Pool of Healthy Donors*

| Leucocyte donor | Tumour cells (TC) | Ratio TC : leucocytes | Incubation with TF $D_{24}$ MI±s.d. | TF $D_{24}$ $P<$ | TF $A_{12}$ MI±s.d. | TF $A_{12}$ $P<$ | No TF MI±s.d. |
|---|---|---|---|---|---|---|---|
| Apparently healthy volunteers | | | | | | | |
| A.E. | B.A. | 1 : 100 | 0·84±0·05 | NS | NT | — | 1·02±0·02 |
| | B.A. | 1 : 50 | 0·68±0·05 | 0·005 | NT | — | 1·00±0·00 |
| | T.G. | 1 : 50 | 0·68±0·03 | 0·025 | NT | — | 1·06±0·06 |
| | B.T. | 1 : 50 | 0·84±0·04 | 0·02 | NT | — | 1·24±0·02 |
| | Pool | 1 : 50 | 0·88±0·02 | NS | NT | — | 1·06±0·06 |
| P.A. | T.A. | 1 : 50 | NT | — | 0·85±0·03 | NS | 0·94±0·07 |
| | B.A. | 1 : 50 | NT | — | 0·77±0·03 | 0·05 | 1·00±0·02 |
| | Pool | 1 : 50 | NT | — | 0·84±0·04 | NS | 1·07±0·07 |
| P.V. | B.A. | 1 : 50 | NT | — | 0·88±0·06 | NS | 1·00±0·05 |
| | T.A. | 1 : 50 | NT | — | 0·77±0·02 | 0·025 | 0·96±0·02 |
| I.L. | B.A. | 1 : 50 | 0·74±0·02 | 0·02 | 0·88±0·04 | NS | 1·00±0·02 |
| | C.R. | 1 : 50 | 0·77±0·03 | 0·02 | 0·91±0·05 | NS | 1·04±0·02 |
| | P.C. | 1 : 50 | 0·62±0·02 | 0·025 | 1·00±0·02 | NS | 0·96±0·04 |
| | Pool | 1 : 50 | 0·91±0·03 | NS | 1·00±0·04 | NS | 1·02±0·02 |
| TCCB patients | | | | | | | |
| B.A. | B.A. | 1 : 200 | 0·75±0·03 | 0·05 | 0·83±0·08 | NS | 1·05±0·05 |
| | B.A. | 1 : 50 | 0·54±0·02 | 0·05 | 0·75±0·01 | 0·05 | 1·10±0·06 |
| | T.G. | 1 : 50 | 0·58±0·02 | 0·025 | 0·77±0·02 | 0·02 | 0·95±0·05 |
| | Pool | 1 : 50 | 0·92±0·11 | NS | 1·00±0·10 | NS | 1·25±0·05 |
| F.B. | F.B. | 1 : 50 | 0·43±0·02 | 0·02 | 0·62±0·05 | NS | 0·71±0·03 |
| | F.B. | 1 : 25 | 0·43±0·03 | NS | 0·50±0·02 | NS | 0·43±0·03 |
| | B.A. | 1 : 50 | 0·71±0·01 | 0·02 | 0·83±0·03 | NS | 1·14±0·06 |
| | B.A. | 1 : 25 | 0·71±0·01 | 0·01 | 0·81±0·01 | NS | 0·86±0·01 |
| | T.G. | 1 : 25 | 0·57±0·03 | NS | 0·75±0·02 | NS | 0·57±0·03 |
| | Pool | 1 : 50 | 1·07±0·07 | NS | 1·00±0·05 | NS | 1·05±0·05 |
| | Pool | 1 : 25 | 1·14±0·04 | NS | 1·00±0·05 | NS | 1·00±0·02 |
| P.G. | P.G. | 1 : 100 | NT | — | 0·80±0·05 | NS | 1·00±0·02 |
| | B.A. | 1 : 25 | NT | — | 0·64±0·01 | 0·02 | 0·77±0·01 |
| | Pool | 1 : 25 | NT | — | 0·90±0·02 | NS | 0·83±0·01 |
| P.C. | P.C. | 1 : 50 | 0·62±0·04 | NS | NT | — | 0·50±0·01 |
| | P.C. | 1 : 25 | 0·19±0·01 | 0·001 | NT | — | 0·44±0·01 |
| | B.A. | 1 : 50 | 1·00±0·02 | NS | NT | — | 1·00±0·02 |
| | T.G. | 1 : 50 | 0·68±0·06 | 0·05 | NT | — | 1·00±0·02 |
| | C.R. | 1 : 50 | 0·68±0·03 | NS | NT | — | 0·75±0·03 |
| | Pool | 1 : 50 | 0·94±0·04 | NS | NT | — | 1·00±0·02 |

Aliquots were incubated with TFdL $D_{24}$ or $A_{12}$, whereas controls were not incubated with TFdL. It is worth noting that transfer of sensitivity was observed in most cases, and when an inhibition was already present prior to the incubation with TFdL, it was increased after TFdL incubation, with the exception of 3 cases. The s.d. was calculated for the MI of each experiment and the $P$ value was obtained by comparing the mean ±s.d. of the MI after TF incubation to the mean ±s.d. of the MI of the same cells without TF incubation.

NT: Not tested.

NS: Not significant ($P > 0 \cdot 05$).

100 ml of tissue culture. In this set of experiments a single induction with TFd was used. After this addition of TFd, the cells were grown for extraction by the addition of fresh medium as required. Samples of approximately $10^8$ cells were taken each week for 4 to 5 weeks and harvested by centrifugation (250 *g* for 10 min.); dialysate was obtained by the same technique used to prepare TFd (Lawrence, 1974). LC not incubated with TFd was also grown, harvested and extracted in the same way. The dialysate thus obtained was used as control and it is designated as Lymphoblastoid Cell Dialysate (LCD).

### RESULTS

Leucocytes from 4 healthy donors and 4 patients with TCCB were tested in LMT against formalin-treated TCCB cells before and after incubation with 2 different TFdL preparations. Formalin-treated TCCB cells from 7 patients (B.A., T.G., P.G., F.B., T.A., C.R. and P.C.) were used for these tests (Table II). It should be noted that the cells from P.G. and F.B. were used only in autologous situations, whereas cells from B.A. P.C. and C.R. were used in both autologous and allogeneic tests. All leucocytes were tested with at least 2 TCCB cell preparations before and after TFdL incubation. Two different TFdLs were used: TFdL $A_{12}$ and TFdL $D_{24}$.

TFdL $A_{12}$ was the product of induction of cell line 43912 (Béchet *et al.*, 1972) with TFd from a patient whose leucocytes were found reactive to TCCB cells from the tumour of patient T.A. TFdL $D_{24}$ was produced by induction of cell line B.F. with TFd from a patient whose lymphocytes were found reactive to tumour cells of patient B.A. and unreactive to T.A. tumour cells.

The results summarized in Table II show that TFdL $A_{12}$ transferred reactivity in 5/14 cases and TFdL $D_{24}$ transferred reactivity in 12/15 cases.

In the autologous situation, i.e. when the patient's leucocytes were tested with his own tumour cells, transfer was obtained with patients B.A. and P.C. using TFdL $D_{24}$, and with patients B.A. and P.G. using TFdL $A_{12}$; the existing reactivity of patient F.B. was increased.

LCD from the uninduced B.F. line used under the same circumstances with the leucocytes of 4 of the TCCB patients who had already shown inhibition when treated with a TFdL preparation, did not increase leucocyte inhibition in the presence of TCCB cells.

Further controls of the specificity of TFdL were performed. It was thus shown that TFdL $A_{12}$ or $D_{24}$ did not transfer any reactivity against formalin-treated cells obtained from 3 melanoma cell lines (Mel-SKI, IGR1, IGR3) and 3 hypernephromata.

Furthermore when cell line B.F. was induced with TFd obtained from patients with hypernephroma or adenocarcinoma of the colon, whose leucocytes had shown reactivity when tested against autologous or homologous tumour cells, the TFdL thus derived failed to sensitize leucocytes obtained from two healthy volunteers tested against formalin-treated TCCB cells.

### DISCUSSION

It has been shown in our laboratory that certain lymphoblastoid cell lines can reproduce TFd extracted from peripheral blood lymphocytes (Viza *et al.*, 1974, 1975). Reactivity to antigens such as PPD, candidine and measles virus can be transferred by this tissue culture TFdL. It is worth noting that although human TFd does not sensitize rats in our hands, as we have already stated in the introduction of this publication, a recent report by some of the co-investigators, whose results claiming a transfer in rats were reported in the very first publication showing TFdL production (Viza *et al.*, 1975), insists that such a sensitization is possible (Goust, Welch and Fudenberg, 1975). The question therefore remains entirely open. The present series of experiments shows that LC can be induced to reproduce TFd from cancer patients sensitized to tumour antigens and this tissue culture TFdL can transfer the tumour sensitivity to non-sensitized leucocytes.

In order to confirm that the dialysate obtained from uninduced LC does not contain any immunological activity, transfer experiments with this dialysate were carried out and they failed to show inhibition in the LMT, whilst the TFdL produced from the same cells was effective.

The absence of inhibition in the LMT, in which hypernephroma or melanoma cells were used as antigens, when a TFdL for TCCB was used for the incubation, suggests the specificity of the TFdL produced *in vitro*. Similarly, the failure of TFdL induced by TFd from non-TCCB cancer patients to sensitize leucocytes to TCCB cells, also indicates specificity of the TFdL. However, the controversy over the specificity transferred by TFd is not settled and it must be emphasized that the purpose of the present report is not to conclude this problem, but merely to show that TFdL after a successful induction behaves in a similar fashion to the TFd used for its production. Nevertheless, it must be admitted that the results obtained are in favour of the specificity of TFd. Further work along these lines using specific TFdL for well-defined, non-tumour antigens, is currently in process and should provide a definite answer.

TFdL was found to transfer reactivity against tumour cells not only to leucocytes from healthy individuals but also to the leucocytes of TCCB—unreactive patients. In 3 cases (B.A., P.G. and P.C.), patients' leucocytes unreactive to autologous tumour cells prior to TFdL incubation were found reactive afterwards. In a fourth patient (F.B.) the reactivity was increased after TFdL incubation (Table II).

Since TFd has already been successfully used for therapy of cancer patients (for recent reviews see Lo Buglio and Neidhart, 1974; Levin *et al.*, 1975; Lawrence, 1974), it is plausible to suppose that in the near future it will be advantageously replaced by TFdL as the latter proved the transfer of sensitivity to tumour cells *in vitro* (Pizza *et al.*, 1975). Indeed, the main restriction of TFd therapy is obtaining large quantities of a TFd with known activity. There are no such limitations for TFdL produced in culture, which can be tested and standardized prior to use.

In the light of the results described here, it seems important for injection of patients to use TFdL capable of sensitizing the patient's leucocytes to the autologous tumour cells now that this can be assessed by the *in vitro* tests. Preliminary results with 3 patients have already shown that TFdL injection transferred the tumour reactivity *in vivo* and the patient's leucocytes became reactive to the autologous and allogeneic tumour cells in the LMT 4 days after the second injection, using TFdL from $10^8$ cells per injection. This reactivity persisted for 6 weeks without further injections. *In vitro* tests performed prior to the TFdL injection had shown that the patient's leucocytes could be rendered reactive to the autologous TCCB cells by the same TFdL used for the subsequent injection of the patient. Further studies aiming for long-term clinical trials with TFdL are now in progress.

The skilful technical assistance of Mrs Avril Collard and Mme Chantal Venet is gratefully acknowledged. The work in Paris was supported by grants from the I.N.S.E.R.M. (ATP 11.74.32), the G.E.F.L.U.C. and the A.R.B.E.

REFERENCES

AX, W. & TAUTZ, C. (1974) Assay of Leucocyte Migration Inhibition under Agarose. *Behring Inst. Mitt.*, **54**, 72.

BÉCHET, J. M., NILSON, K. & KLEIN G. (1972) In *Oncogenesis and Herpes Virus*. W.H.O. Intenational Agency for Research on Cancer: Lyon. p. 249.

BLOOM, E. T., OSSORIO, R. C. & BROSMAN, S. A. (1974) Cell Mediated Cytotoxicity against Human Bladder Cancer. *Int. J. Cancer*, **14**, 326.

BUBENIK, J., PERLMANN, P., HELMSTEINEN, K. & MOBERGER, G. (1970) Immune Response to

Urinary Bladder Tumours in Man. *Int. J. Cancer*, **5**, 39.

GOUST, J. M., WELCH, M. & FUDENBERG, H. H. (1976) Human " Transfer Factor " Activity in Guinea Pigs. In *2nd Workshop on Basic Properties and Clinical Application of Transfer Factor*, London and New York: Academic Press. (In press)

LAWRENCE, H. S. (1974) Transfer Factor in Cellular Immunity. *The Harvey Lecture Series 68*. London and New York: Academic Press. p. 239.

LEVIN, A. S., BYERS, V. S., FUDENBERG, H. H., WYBRAN, J., HACKETT, A. J., JOHNSTON, J. O. & SPITLER, L. E. (1975) Osteogenic Sarcoma: Immunologic Parameters Before and During Immunotherapy with Tumour-specific Transfer Factor. *J. clin. Invest.*, **55**, 487.

LO BUGLIO, A. F. & NEIDHART, J. A. (1974) A Review of Transfer Factor Immunotherapy in Cancer. *Cancer*, **34**, 1563.

O'TOOLE, C., PERLMANN, P., WIGZELL, M., UNSGAARD, B. & ZETTERLUND, C. C. (1973) Lymphocyte Cytotoxicity in Bladder Cancer—No Requirement for Thymus Derived Effector Cells? *Lancet*, i, 1085.

PIZZA, G., VIZA, D., BOUCHEIX, CL. & CORRADO, F. (1976) Studies with *In Vitro* Produced Transfer Factor. In *2nd Workshop on Basic Properties and Clinical Applications of Transfer Factor*. London and New York: Academic Press. (In press)

ROSS, C. E., COCHRAN, A. J., HOYLE, D. E., GRANT, R. M. & MACKIE, R. M. (1973) Formalin-fixed Tumour Cells in the Leukocyte Migration Test. *Lancet*, ii, 1087.

SEGALL, A., WEILER, O., GENIN, J., LACOUR, J. & LACOUR, F. (1972) *In Vitro* Study of Cellular Immunity against Autochthonous Human Cancer. *Int. J. Cancer*, **9**, 417.

VIZA, D., GOUST, J.-M., MOULIAS, R. & MÜLLER-BÉRAT, N. (1974) *In Vitro* Production of Transfer Factor. *Scand. J. Immunol.*, **3**, 892.

VIZA, D., GOUST, J.-M., MOULIAS, R., TREJDOSIEWICZ, L. K., COLLARD, A. & MÜLLER-BÉRAT, N. (1975) *In Vitro* Production of Transfer Factor by Lymphoblastoid Cell Lines. *Transplant. Proceedings*, **8**, Suppl. I, 329.